# Feasibility of the Maastricht Innovation Readiness Approach: a self-assessment of innovation readiness in long-term care organizations for older adults

**DOI:** 10.3389/frhs.2026.1660216

**Published:** 2026-04-30

**Authors:** Monique W. van den Hoed, Ramona Backhaus, Erica de Vries, Jan P. H. Hamers, Ramon Daniëls

**Affiliations:** 1 Department of Health Services Research, Faculty of Health, Medicine and Life Sciences, CAPHRI Care and Public Health Research Institute, Maastricht University, Maastricht, Netherlands; 2 Living Lab in Ageing and Long-Term Care, Maastricht, Netherlands; 3 Fliedner Fachhochschule Düsseldorf, Düsseldorf, Germany; 4 Zuyd University of Applied Sciences, Expertise Centre for Innovative Care and Technology, Research Centre for Assistive Technology in Health Care, Heerlen, Netherlands

**Keywords:** consensus meeting, feasibility, framework, innovation maturity, innovation readiness, long-term care, MIRA, self-assessment

## Abstract

**Background:**

Innovation in long-term care for older adults is essential to manage challenges such as a growing demand and workforce shortages. Organizations with higher innovation readiness are more likely to adopt and sustain innovations effectively. However, an approach indicating the innovation readiness of long-term care organizations for older adults is lacking. To address this, the Maastricht Innovation Readiness Approach (MIRA) was developed to (1) increase understanding about innovation readiness, (2) facilitate the organization's self-assessment of innovation readiness, and (3) inspire the organizational conversation on how to become better at innovating.

**Objective:**

The aim of this work was to examine the feasibility of MIRA and explore whether it could be successfully applied in long-term care practice.

**Methods:**

MIRA consists of a questionnaire and a consensus meeting. To evaluate the MIRA approach, a mixed-method cross-sectional feasibility study was conducted in 10 Dutch long-term care organizations. The study evaluated scores on the MIRA Questionnaire, assessed its intrarater reliability, and examined the feasibility of both the MIRA Questionnaire and the MIRA Consensus meeting. Professionals (involved in innovation) were asked to (1) complete the MIRA Questionnaire and additional closed-ended questions about its feasibility twice online (1-month interval), (2) participate in the Consensus meeting and verbally answer open questions, and (3) complete a questionnaire directly following the consensus meeting on its feasibility.

**Results:**

In total, 173 participants completed the MIRA Questionnaire at *t*1 (#128 at *t*2); 127 participants attended the MIRA Consensus meeting (*t*1). The mean perceived innovation readiness score was 6.6 (scale 1–10). Intrarater reliability of the MIRA Questionnaire was good (intraclass correlation coefficient > 0.75). Participants evaluated MIRA as acceptable, suitable, and valuable: 88% indicated that MIRA provided insight into their organization's innovation readiness, and 84% would recommend it to other organizations. Interestingly, participants suggested annual use of MIRA.

**Conclusion:**

MIRA is a feasible approach to assess innovation readiness in long-term care. It enhances internal awareness, supports group reflection across roles and disciplines, and may support structured progress in innovation readiness. However, it remains unclear whether conducting MIRA improves innovation readiness. Longitudinal studies are needed to follow organizations as they implement steps to improve innovation readiness and to explore how these contribute to succesful innovation outcomes.

## Introduction

Innovation in long-term care for older adults is crucial to address challenges such as growing demand, workforce shortages, and shrinking resources (1). Greenhalgh et al. (2) define innovation as “a novel set of behaviors, routines, and ways of working that are discontinuous with previous practice, are directed at improving health outcomes, administrative efficiency, cost-effectiveness, or user experience, and that are implemented by planned and coordinated actions.” Organizations that are more innovation ready adopt innovations more swiftly and sustain them more durably (3–5). “Innovation readiness” indicates an organization's maturity level to succeed in implementing innovation and the extent to which it has organized and prepared key factors to facilitate innovation readiness (6).

Long-term care organizations are insufficiently aware of the competencies and preconditions needed for successful innovation (6–8). To become better at innovating, organizations must actively work on innovation readiness. Understanding the organization's current state is the first step toward improvement (9–11). By gaining insight into factors that contribute to becoming better at innovating, combined with assessing their level of innovation readiness maturity, provide organizations with a structured basis to identify and prioritize opportunities to strengthen their innovation readiness (12–14).

Various assessments and instruments have been developed to assess readiness to implement a specific innovation (15–17). Instruments assessing innovation readiness were either not scientifically developed, evaluated, or tested in health care (18–21). An approach indicating the innovation readiness of long-term care organizations for older adults is lacking (22–24). To address this gap, we developed the Maastricht Innovation Readiness Approach (MIRA) in collaboration with an advisory group of managers and healthcare professionals involved in innovating within long-term care organizations (25). The purpose of MIRA is to (1) increase understanding about innovation readiness, (2) facilitate the organization's self-assessment of innovation readiness, and (3) inspire organizational dialogue on how to become better at innovating. The MIRA Framework (Supplementary Figure S1) was constructed based on the results of a scoping review of scientific studies on the innovation readiness of healthcare organizations (6), supplemented by qualitative research (26–28) and confirmed by a Q-study (29) in long-term care. Based on the MIRA Framework, we iteratively created and refined the MIRA Questionnaire, which was tested for content validity and piloted (Table 1) (30–32). Finally, a structured MIRA Consensus meeting (Supplementary Table S1) was designed to collectively interpret the questionnaire results.

**Table 1:** MIRA Questionnaire.

Questions (#31) in four innovation readiness domains: (1) strategic direction, (2) organization of innovation, (3) leadership for innovation, and (4) learning climate
*Strategic direction for innovation (M)*
**1. In my organization, innovation is guided by our ambition.**
Innovation ambition includes what kind of innovation the organization pursues and why (improving, renewing, and/or radical innovation) and the associated ambition (e.g., being a follower or leader).
**2. My organization ensures that innovation themes provide direction for innovation.**
Innovation themes: areas in which you want to achieve added value through innovation, for example, sustainable employability of staff or enhancing autonomy for clients.
**3. In my organization, we use an annual plan for innovation.**
An innovation plan with a budget, actions, and strategies to execute the innovation ambition step by step.
**4. In my organization, the innovation budget is adjusted where necessary.**
Innovation budget: funds allocated for innovation, including the purchase of innovations and time for employees dedicated to innovation projects.
**5. My organization has defined specific tasks, roles, and positions for innovation.**
The organization describes innovation tasks and responsibilities per position and/or team.
**6. My organization ensures that the technical infrastructure supports innovation.**
Technical prerequisites are in place to support all types of innovations (social, process, technological).
**7. My organization communicates about innovation to employees.**
Planned communication about, for example, innovation ambition, projects, experiences, and results.
*Organization of innovation (M)*
**8. My organization visibly pays attention to the organization of innovation.**
Innovation is visibly organized by, for example, establishing innovation roles, teams, and committees specifically focused on this.
**9. My organization ensures there is an overview and insight into the progress of innovation.**
Having an overview of innovation projects to guide decision-making, for example, through progress reports.
**10. My organization uses a specific approach to shape the innovation process.**
This approach involves the steps in an innovation process, such as how to start an innovation project, how long it will take, and how to implement it.
**11. My organization uses a toolbox with innovation methods.**
A collection of innovation techniques and tools, such as a template for an implementation plan or brainstorming approaches.
**12. My organization ensures that decision-making supports innovation.**
There is clarity within the organization about who decides what and when in the innovation process, including prioritizing innovation themes, project selection, evaluation, progress, and budget.
**13. My organization involves clients, family members, and close contacts in innovation.**
Clients and their relatives provide information or advice or contribute ideas for innovations.
**14. My organization provides opportunities to employees to be involved in innovation.**
The organization gives all employees the opportunity to participate in innovation, both solicited and unsolicited, for example, by working on their own ideas.
**15. My organization exchanges knowledge and experience on innovation with healthcare and knowledge institutions.**
Sharing knowledge and experience, such as experiences with implementation and lessons learned.
**16. My organization keeps track of regional and national developments in the field of innovation.**
Monitoring developments in society, government, health insurance offices, suppliers, and participating in events that offer opportunities for innovation in your organization.
**17. My organization collaborates with companies in the field of innovation.**
Collaborating with, for example, ICT companies and suppliers of innovations by, among others, providing feedback on new products and conducting experiments, pilots, and implementations.
**18. My organization shapes learning from/about innovation based on a vision.**
A vision that indicates how the organization learns from/about innovation and how the organization creates a work environment that promotes learning about innovation.
*Leadership for innovation (M)*
**19. In my organization, the board expresses that innovation is a priority.**
The board lets employees know and feel that innovation is important, for example, by continuing to support innovation initiatives even when things “go wrong.”
**20. My organization clearly expresses to managers what is expected of them in terms of innovation.**
For managers, expectations in the area of innovation are clear, such as encouraging employees, presenting their own innovation plans, and creating a supportive environment.
**21. My organization invites employees to participate in innovation.**
Employees are encouraged and know what is expected regarding innovation. For example, bringing up own ideas, pointing out bottlenecks, voicing doubts, and daring to try something new.
**22. In my organization, managers actively involve employees in the innovation process.**
Managers give employees space to engage in innovation, for example, by translating innovation policies into opportunities for the team and translation of the team's wishes to the organization.
*Learning climate (M)*
**23. My organization ensures that innovation knowledge is available and accessible for everyone.**
The approach to innovations, the experiences, and the results from innovation projects are shared within the organization, for example, knowledge of what went well and what did nt.
**24. My organization ensures alignment between strategy, organization, leadership, and learning environment to improve in the field of innovation.**
To get better at innovating, alignment between the organization and the execution of innovation is important, for example, by detailing the innovation ambition into innovation themes to support decision-making.
**25. My organization stimulates knowledge exchange between employees in the field of innovation.**
Facilitating moments and meetings to exchange experiences related to innovation.
**26. My organization evaluates progress in getting better at innovating.**
Together, it is discussed whether the steps that have been taken to improve have been successful and why.
**27. My organization uses research to support and evaluate innovations.**
Research conducted by the organization or by others is used to develop innovations and to evaluate whether the intended effect has been achieved.
**28. In my organization, we have a training plan for innovation.**
A plan and schedule for training in the field of innovating, for example, which courses and trainings and for whom.
**29. My organization offers opportunities for employees to receive training in the field of innovation.**
Support and training for employees, for example, in implementing innovation in their own work environment or shaping a brainstorming session.
**30. In my organization, managers are trained to stimulate innovation within their teams.**
Training for managers to facilitate innovation within their teams, for example, how to identify a need for innovation.
**31. My organization provides rooms for innovation activities.**
Specific rooms or locations are set up for innovation activities, such as trainings, brainstorming sessions, and team meetings.

We sought to examine whether MIRA could be successfully applied in long-term care practice. This study aimed to examine the perceptions of long-term care professionals regarding the feasibility of MIRA in Dutch long-term care organizations and explore whether MIRA has the potential to support innovation readiness in this context. The research was guided by the following research questions: (1) How do participants score the MIRA Questionnaire and rate their organization's innovation readiness? (2) What is the intrarater reliability of the MIRA Questionnaire? (3) How do participants evaluate the feasibility of the MIRA Questionnaire and Consensus meeting?

## Materials and methods

### Study design

This study applied a convergent mixed-method cross-sectional design (30, 31) and was conducted in long-term care organizations in the Netherlands between July 2024 and December 2024.

### Participants and setting

MIRA was developed for long-term care organizations for older adults in the Netherlands (6, 28, 29) (e.g., care homes, nursing homes, assisted living facilities, and residential aged care). These organizations provide a range of services such as medical, transitional, and nursing care, housing, personal care, assistance, and social services to older adults who are unable to live independently (32). Dutch long-term care is largely funded through mandatory public health insurance and increasingly invests in innovations such as digital technologies, new care models, and organizational redesign (33). While some larger organizations have dedicated innovation managers or teams, many long-term care organizations rely on project-based structures or multidisciplinary working groups to develop and implement innovations.

We intended to test the MIRA Questionnaire and the MIRA Consensus meeting in 10 long-term care organizations, which were approached and selected through purposive sampling based on their capacity and interest in implementing MIRA. Five were recruited through employers' organizations for care and welfare, two through the Living Lab in Ageing and Long-Term Care, and three through the research team's network (34). We instructed the internal coordinator (who was appointed by the participating organization) to include healthcare professionals who were involved in innovation and had sufficient insight to reflect on their organization's innovation readiness, ideally representing a range of roles, disciplines, and organizational levels. Participating organizations were required to invite a minimum of 10 participants to complete the MIRA Questionnaire and a minimum of eight participants to attend the MIRA Consensus meeting.

### Data collection

#### MIRA

Data were collected using the MIRA Questionnaire and the MIRA Consensus meeting. The MIRA Questionnaire contains 31 questions in four domains: (1) strategic direction, (2) organization of innovation, (3) leadership for innovation, and (4) learning climate. Participants assessed factors contributing to their organization's innovation readiness. Each question used the same six scoring options (ordinal) (Table 2): “not,” “informal,” “occasionally,” “consistently,” “optimally,” and “no insight.” These categories were informed by innovation maturity scales used in business (21, 35). These maturity positions (Table 2) outline an organization's path to achieving innovation readiness. They consist of the three interconnected elements of frequency, agreements, and learning: (1) frequency is the extent to which organizations have organized the innovation readiness factors; (2) agreements involve to whether agreements have been made (e.g., in internal sessions or policies of the organization), and (3) learning refers the extent to which organizations reflect on and learn from initiatives to improve innovation readiness. The frequency of agreement execution indicates the extent to which innovation-related actions are consistently implemented within an organization's day-to-day operations (36). Embedding innovation through agreements helps sustain strategic focus and integrates innovation into the organization's long-term learning (36). The component of learning helps to turn individual insights into collective capabilities and embed innovation into daily practices (36, 37).

**Table 2:** Innovation readiness maturity positions: response options.

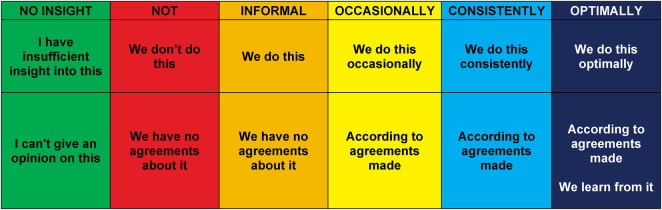

#### Quantitative data collection

The MIRA Questionnaire was distributed to participants via the online survey platform Qualtrics, following an email invitation from the internal coordinator. Participants completed the questionnaire on either a desktop or mobile device and received a summary of their responses by email upon completion. Automatic reminders were sent after 7 days to those participants who had not yet completed the questionnaire. Participants were individually asked for consent and briefed on data use.

The MIRA Consensus meeting (38) followed a predefined set-up and interview guide (Supplementary Table S1) and was moderated by researcher MH. During the meeting, background information on innovation readiness was given, and the questionnaire results were presented. The meeting lasted 2 h and was held within 2 months after completing the MIRA Questionnaire at the location of the long-term care organization.

#### Scores on innovation readiness

Participants’ scores on innovation readiness were collected via the MIRA Questionnaire (Table 1). One extra question was added to the MIRA Questionnaire (at *t*1), asking the participants to rate their organization's innovation readiness on a scale from 1 to 10 (to complement the overall picture of the participating organizations).

#### Intrarater reliability data

To evaluate inter-rater reliability, participants completed the MIRA Questionnaire twice, at both *t*1 and *t*2.

### Qualitative data collection

#### Feasibility dimensions

To evaluate the feasibility of the MIRA Questionnaire and the MIRA Consensus meeting, we applied the feasibility dimensions described by Gadke et al. and Orsmond et al. (38, 39): acceptability, suitability, and added value [Table 3, including the related research questions and operationalization (40)]. Acceptability refers to how participants perceive and respond to MIRA, including whether it is agreeable, satisfactory, or easy to use (38, 39, 41). Suitability refers to the perceived fit or relevance of MIRA for a particular setting (39, 42, 43). Added value indicates the perceived usefulness, that is, the extent to which participants find MIRA contributes to innovation readiness (39, 42).

**Table 3:** Feasibility dimensions.

Feasibility dimensions	Research question	Operationalization
Acceptability and suitability		
MIRA questionnaire		
1a. show that the MIRA Questionnaire is appropriate for use by participants	How do participants evaluate the acceptability and suitability of the questionnaire?	The questions were clear.^a^^,^^c^
		The response options were clear.^a^^,^^c^
		The instructions on the first page were sufficient to complete the questionnaire.^a^^,^^c^
		How did you experience answering these questions?^b^
		Did answering these questions benefit you personally?^b^
		Do you have any questions or comments about the questions asked?^b^ *(questions were part of the online MIRA Questionnaire at t1)*
MIRA Consensus meeting		
1b. show that the MIRA Consensus meeting is appropriate for use and appealing to participants	How do participants evaluate the acceptability and suitability of the consensus meeting?	The composition of the group of participants in the consensus meeting was appropriate.^a^
		The feedback of the results of the MIRA questionnaire was understandable.^a^
		I liked the set-up of the consensus meeting.^a^ *(questions were answered on paper in the MIRA Consensus meeting)*
Added value		
MIRA questionnaire & consensus meeting		
2. show that the participants experience the use of MIRA as relevant	How do participants evaluate the added value of MIRA (questionnaire and consensus meeting)?	Due to the consensus meeting, we know better where we stand in terms of innovation readiness.^a^
		I now have more insight into factors that are important for innovation readiness.^a^
		I would recommend this approach (questionnaire plus consensus meeting) to other organizations.^a^
		Our organization could have conducted the questionnaire and led the consensus meeting (without external support).^a^ *(questions were answered on paper in the MIRA Consensus meeting)*
		How did you experience MIRA (questionnaire and consensus meeting)?^b^ *(question asked by researcher MWH at the end of the consensus meeting)*

a Five-Point Likert scale: fully disagree, disagree, neither agree nor disagree, agree, fully agree.

b Open-ended question.

c Data available for six out of 10 organizations (due to administrative reasons).

Data for evaluating the dimensions of “acceptability” and “suitability” of the MIRA Questionnaire were collected at *t*1 by means of six additional questions (three closed-ended and three open-ended).

To evaluate the “added value” of MIRA (Questionnaire and Consensus meeting) and the “acceptability and suitability” of the MIRA Consensus meeting, participants answered closed-ended questions on paper at the end of the meeting.

During the consensus meeting, MH invited participants to actively reflect on the results (recognizability of the results, similarities and differences in the innovation readiness scores), explore opportunities toward innovation readiness, and share their opinions on MIRA's “added value.” Researchers KD, LJ, EV, MD, and MH observed the meeting and documented participant reflections.

### Quantitative data analysis

#### Scores on innovation readiness

The innovation readiness scores for each organization overall, per domain and question separately were calculated by summing all participants scores for all 31 questions, per domain and question, respectively, without weights (each score was given equal weight) (39). Results were presented as percentages (of the innovation readiness maturity scoring options). MIRA Questionnaires with missing data were excluded from the analysis. The numeric ratings on the perceived innovation readiness score (scale of 1–10) were summed and divided by the number of participants to calculate the mean for each organization.

#### Intrarater reliability data

For the intrarater reliability (to evaluate “participant reporting”), the intraclass correlation coefficient (ICC) was used (via SPSS). Estimates above 0.75 were considered as good, between 0.50 and 0.75 as moderate, and below 0.50 as poor reliability scores (40). A good score on intrarater reliability means that the questionnaire has low variability in interpretation, indicating that questions and scoring options are clear and well understood.

### Qualitative data analysis

#### Feasibility dimensions

The closed-ended questions (5-point Likert scale) were analyzed in SPSS. Descriptive statistics were calculated, including percentages, means, and standard deviations. Mean scores (for the Likert scale) ranging between 1.0 and 2.4 were considered inadequate, between 2.5 and 3.4 as needing attention, between 3.5 and 3.9 as acceptable, between 4.0 and 4.4 as good, and 4.5 and 5.0 as very good (41). Likert scale responses were treated as ordinal data and summarized using descriptive statistics.

Thematic content analysis was employed for the open-ended questions. To analyze the group reflection on MIRA's “added value,” researcher MH reviewed the observation notes and conducted a narrative summary of the recurring themes. Questionnaires with missing data were excluded from the analysis.

#### Research ethics

The study received ethical approval from the Medical Ethics Board of Zuyderland Medical Center in the Netherlands, with the approval number METCZ20220036. The data collection and data storage plan were approved by the local General Data Protection Regulation committee of the University of Maastricht.

Furthermore, participating organizations and their staff were informed about the study's ethical approval, objectives, the voluntary nature of participation, and that they could withdraw at any time without consequences. Each organization was asked to obtain consent from the Board of Directors to participate in this study.

Participants were informed via an invitation e-mail and at the beginning of the MIRA Questionnaire (page 1). By proceeding to the second page with the substantive questions, participants provided consent. The internal coordinators made their own decisions about which participants to include for the MIRA Questionnaire and the MIRA Consensus meeting.

As the study focused on organizational processes rather than personal health information, no significant risks were anticipated. Confidentiality was safeguarded through anonymized and aggregated reporting at the organizational level and through secure data storage in compliance with General Data Protection Regulation (GDPR) (42).

## Results

The MIRA Questionnaire and the MIRA Consensus meeting were tested in 10 long-term care organizations that varied in size and were geographically spread across the Netherlands. Participants from different organizational levels were invited by the participating organizations: board members; care directors; innovation managers; staff responsible for finance, policy, information and communication technology (ICT), communication, facilities, and quality with a role in innovation; client representatives; team managers; nursing home managers; medical and wellbeing staff; human resources managers and staff involved in learning; direct care professionals, both in residential long-term care and community-based care, such as registered nurses—baccalaureate-educated and vocationally trained; and certified nurse assistants with a role in innovation.

Two related but analytically distinct samples were included. The quantitative sample consisted of 173 participants at *t*1 and 128 participants at *t*2 who completed the MIRA Questionnaire (Table 1) [including the question about the numeric rating (1–10) on the organization's innovation readiness]. Eligible participants were professionals with insight into their organization's innovation processes, invited via an internal coordinator. The qualitative sample consisted of participants attending the MIRA Consensus meetings. At least eight participants were required from each organization; the actual number of attendees ranged from 8 to 38 per organization, with a total of 127 participants across all meetings (of which 113 completed the closed-ended questions on paper at the end of the consensus meeting). Although many consensus meeting participants had also completed the MIRA Questionnaire, participation in both components was not mandatory and samples partially overlapped. Due to administrative reasons, only 103 participants completed the open-ended questions on the acceptability and suitability of the MIRA Questionnaire at *t*1.

### Scores on innovation readiness

Table 4 presents participants' ratings of their organization's innovation readiness. This includes both the numeric rating (on a scale from 1 to 10), reflecting the overall perceived level of innovation readiness, and the aggregated scores derived from the 31 items of the MIRA Questionnaire. The aggregated scores represent the combined responses of all participants within each organization, providing an overall picture of innovation readiness per participating organization.

**Table 4:** Participants MIRA questionnaire scores—#10 LTC organizations.

No.	Size of LTC^a^ organization^c^	SD	Numeric rating of innovation readiness^d^ (mean)	Overall innovation readiness score (#31 MIRA questions summed up)					
				No insight	Not	Informal	Occasionally	Consistently	Optimally
	Turnover in euro's			*I have insufficient insight into this*	*We don't do this*	*We do this*	*We do this occasionally*	*We do this consistently*	*We do this optimally*
				*I can't give an opinion on this* (%)	*We have no agreements about it* (%)	*We have no agreements about it* (%)	*According to agreements made* (%)	*According to agreements made* (%)	*According to agreements made*
									*We learn from it* (%)
1	Large^b^	0.9	6.4	14	8	10	26	34	8
2	Medium^b^	0.8	7.2	8	12	14	20	37	9
3	Small^b^	1.7	6.0	9	11	21	22	24	13
4	Medium^b^	1.4	5.7	11	16	21	24	24	4
5	Large^b^	1.4	6.6	22	10	10	21	29	8
6	Medium^b^	1.3	7.3	15	6	12	24	30	13
7	Medium^b^	0.6	6.6	15	2	9	21	35	18
8	Medium^b^	1.0	6.3	6	8	15	28	37	6
9	Medium^b^	1.1	7.4	9	9	9	27	34	12
10	Medium^b^	1.1	6.6	6	5	12	27	29	21
Mean		1.1	6.6	12	9	13	24	31	11
Range		1.1 (0.6–1.7)	1.7 (5.7–7.4)	16 (6–22)	11 (5–16)	12 (9–21)	8 (20–28)	8 (29–37)	14 (4–18)

a Long-term care organization for older adults providing medical, transitional, and nursing care, housing, personal care, assistance, and social services to older adults who cannot live independently.

b Annual reports 2024.

c Turnover in euros: small €0–100 M, medium €100–200 M, large €200 M and more.

d Numeric rating—all things considered, what rating would you give your organization in terms of innovation readiness (1–10)?

### Intrarater reliability data

A total of 128 participants completed the MIRA Questionnaire at *t*1 and *t*2, about a month apart. The overall 1-month intrarater reliability was good (ICC above 0.75) (Table 5). The ICC values for the intrarater reliability per question (presented with a 95% confidence interval in the Supplementary Additional File and at https://osf.io/x73p6/files/hqv4n) ranged from “moderate” to “good.” Participants gave the same answers at *t*1 and *t*2 for 38%–63% of the questions. For nine specific questions (questions 7, 11, 12, 14, 15, 16, 22, 25, and 30), at least half of the participants gave the same answer both times. The proportion of questions with stable or only slightly changed responses—defined as the same score or a one-step difference between t1 and t2 (e.g., ‘occasionally’ at t1 and ‘consistently’ at t2, or one step lower) - ranged from 73% (Table 1, question 4) to 88% (Table 1, question 8). This indicates that, for most questions, participants' ratings remained largely consistent over time, with only minor variations between measurement moments.

**Table 5:** Intrarater reliability MIRA questionnaire (ICC estimates, 95% CI, absolute-agreement, 2-way mixed-effects model) (https://osf.io/x73p6/files/hqv4n: ICC for all 31 MIRA questions (see Supplementary Additional File).

Response option	*t*1 (%)	*t*2 (%)	Difference (%)	ICC (intraclass correlation) value	95% confidence interval	
					Lower bound	Lower bound
Not	9.2	8.5	−0.7	Single 0.992	Single 0.947	Single 0.999
Informal	14.8	14.9	0.1			
Occasionally	23.4	23.2	−0.2			
Consistently	30.1	32.2	2.1	Average 0.996	Average 0.996	Average 0.999
Optimally	12.3	11.5	−0.8			
No insight	10.3	9.8	−0.5			

### Feasibility dimensions

#### Acceptability and suitability of the MIRA Questionnaire

Participants rated the questions and the response options overall as clear (>72% agree and fully agree). The instructions in the cover letter were regarded as sufficient to complete the questionnaire (92% agree and fully agree) (Table 6). The results showed that participants evaluated the Questionnaire positively across several aspects of usability. They experienced the language as suitable and easy to understand, the routing and navigation as logical, and the instructions as clear. In addition, the response options were perceived as intuitive, and the overall design of the online presentation—including the use of color to distinguish the six scoring options—was appreciated. It showed that participants rated the questionnaire positively for its language use (suitable), routing and navigation (logical), instructions (clear), response options (intuitive), and the design of the online presentation (including the colorful design of the 6 scoring options). Some participants mentioned that they selected the option “no insight” due to limited information or overview to answer specific questions related to their role or position in the organization. Furthermore, in the open-ended questions, participants provided suggestions for improvement on the cover letter (emphasizing in instructions that the focus should reflect the entire organization, and not a specific department), on the term innovation (clarifying the term), and on the design (adding an option at each question to write a rationale or explanation). Furthermore, participants found the term “agreements” in the scoring options to be vague. They suggested clarifying what qualifies as an agreement, whether it refers to informal discussions or documented policies. For question 28, participants suggested clarifying whether “the training plan” refers generally to education about innovation or to the existence of a specific training plan within an innovation project. For question 31, they proposed adding “the possibility to organize room for innovation activities.”

**Table 6:** Acceptability and suitability of the MIRA questionnaire.

Closed and open-ended questions (MIRA Questionnaire at t1)	Mean (5-point Likert scale)	Median	SD	% Agree/ fully agree
Closed questions (MIRA Questionnaire at *t*1)				
The questions were clear^a^	3.7	4.0	0.65	73
The response options were clear^a^	3.7	4.0	0.74	72
The instructions on the first page were sufficient to complete the questionnaire^a^	4.2	4.0	0.56	92
Open-ended questions (MIRA Questionnaire at *t*1)				
How did you experience answering these questions?	Data available (43) https://osf.io/x73p6/			n.a.
Did answering these questions benefit you personally?				n.a.
Do you have any questions or comments about the questions asked?				n.a.

a Data from six out of 10 organizations.

On average, the self-assessment took 10–15 min to complete, which was considered acceptable. Participants felt that the time investment was manageable within their daily workflow. The MIRA Questionnaire was perceived by the participants as relevant to their work, as it helped them reflect on their role in this process, increased their understanding of innovation readiness, and provided insight into their organization's innovation maturity. Furthermore, the questions and structured scoring options provided awareness of the next steps the organization needed to take toward innovation readiness.

#### Acceptability and suitability of the MIRA consensus meeting

Participants rated the feedback on the results of the questionnaire (90% agree/fully agree) and the set-up of the consensus meeting as good (92% agree/fully agree) (Table 7). During the consensus meeting, participants indicated that reflecting on the MIRA results increased the practical value and impact of the approach, as it supported the journey to become innovation ready. Some participants indicated at the end of the meeting that the insights they now have into innovation readiness would lead to more positive scores on the MIRA Questionnaire. Furthermore, participants stated that the diversity of attendees was of value for the discussion. They were positive about the group reflection and discussion on innovation readiness, in particular when colleagues joined the consensus meeting whom they had never met before or discussed innovation readiness with. When participants indicated that the composition of the consensus meeting was not appropriate, they most often noted that senior management was not sufficiently represented or that more healthcare professionals should have been involved, given the importance of the topic.

**Table 7:** Acceptability and suitability of the MIRA consensus meeting.

Closed-ended questions	Mean (5-point Likert scale)	Median	SD	% Agree/fully agree
The composition of the group of participants in the consensus meeting was appropriate	3.9	4.0	0.72	78
The feedback of the results of the MIRA Questionnaire was understandable	4.3	4.0	0.60	90
I liked the set-up of the consensus meeting	4.3	4.0	0.57	92

#### Added value of MIRA questionnaire and consensus meeting

Participants rated the added value of both the MIRA Questionnaire and the MIRA Consensus meeting as good (>80% agree and fully agree) (Table 8). Seventy-seven percent indicated that extra (external) assistance is needed for conducting the consensus meeting. During the consensus meetings, participants expressed interest in repeating MIRA annually to monitor their innovation readiness maturity. Furthermore, they said that to accelerate learning, they would like to discuss innovation readiness with other long-term care organizations. At all 10 MIRA Consensus meetings, organizations discussed possible next steps toward innovation readiness.

**Table 8:** Added value of MIRA questionnaire and consensus meeting.

Closed questions	Mean (5-point Likert scale)	Median	SD	% Agree/fully agree
Consensus meeting				
Due to the consensus meeting, we know better where we stand in terms of innovation readiness	4.2	4	0.63	88
Consensus meeting and Questionnaire				
I now have more insight into factors that are important for innovation readiness	4.1	4	0.66	84
I would recommend this approach (questionnaire plus consensus meeting) to other organizations	4.1	4	0.66	84
Our organization could have conducted the questionnaire and led the consensus meeting (without external support)	2.7	3	1.15	23

## Discussion

The goal of this study was to examine the perceptions of long-term care professionals regarding the feasibility of the MIRA in Dutch long-term care organizations. The objective was also to evaluate the MIRA approach, which combines a framework, questionnaire, and consensus meeting to assess and facilitate innovation readiness.

The main findings show that long-term care professionals perceived MIRA as useful and relevant for enhancing their organization's innovation readiness. The participants were positive about both insights into organizational innovation readiness maturity and the appropriateness for use. The time it took to complete the MIRA Questionnaire was considered acceptable, and the “good” intrarater reliability indicates that the questions and scoring options were clear and well understood. Another important finding is that participants emphasized the relevance of the MIRA Consensus meeting. They appreciated discussing the results of their organization's innovation readiness maturity in the presence of a broad selection of healthcare professionals. Innovation readiness concerns a diversity of functions in the organization (6), and participants valued the opportunity to discuss the subject with colleagues with whom they rarely interacted. Tidd et al. (36) highlighted that reflecting on results together (rather than individually) may foster a shared understanding of an organization's innovation enablers and barriers. In addition, Haraldseid et al. (43) showed that group reflection supports collaborative learning, especially creating space for reflection enhances individual, team, and organizational learning (44). According to Halcomb et al. (44), a meaningful consensus meeting combines available information and facilitates the consensus process between the participants, thereby increasing their ownership and engagement. Altogether, the MIRA Consensus meeting provided insight into each organization's innovation readiness, identified where the organization's opportunities for innovation readiness lie, and supported gradual progress in building innovation readiness in a structured way.

Interestingly, all organizations rated themselves as having ‘modest’ innovation readiness, suggesting a cautious self-assessment. Across organizations, the response option ‘consistently’ was most frequently selected for overall innovation readiness, and the mean numeric rating for perceived innovation readiness was 6.6. These scores may suggest that professionals across the 10 organizations were careful and nuanced in assessing the innovation readiness of their organizations. Larsson et al. (45) discovered that moderate scoring is not uncommon in organizational evaluations, particularly in complex domains, where participants in certain roles may lack full visibility into all relevant aspects, leading them to give more cautious or moderate ratings (9, 46). In our earlier study, we showed (29) that stakeholders in long-term care organizations often have varying perspectives on innovation readiness, influenced by their roles and access to information, which might lead to a flattened score. Overall, our findings suggest that the modest innovation readiness scores may reflect not a lack of readiness *per se*, but rather the complexity of innovating in practice and variation in perspectives across organizational roles and levels.

This raises the question of whether MIRA, as a self-assessment instrument, should be able to distinguish between organizations with different levels of innovation readiness. Self-assessment instruments, such as MIRA, may face limitations in precisely differentiating innovation readiness levels; their main added value may be found when used for reflection or development, rather than as an instrument for producing comparative scores (47, 48). Innovation readiness is context-dependent, shaped by factors such as an organization's strategic direction, leadership, and learning climate. These factors differ between organizations, and what is considered “ready” in one context may not be sufficient in another. Consequently, innovation readiness should be viewed and interpreted as a situational assessment, rather than as an objective benchmark. To encourage unbiased responses, it is important to emphasize that MIRA, as a subjective self-assessment of an organization's innovation readiness, is not designed for benchmarking purposes. This underscores the importance of combining the MIRA Questionnaire with structured dialog in the MIRA Consensus meeting to substantiate scores and expand participants’ shared understanding.

Participants valued both the MIRA Questionnaire and the MIRA Consensus meeting. However, while all organizations expressed intentions to improve their innovation readiness, it remains unclear whether and to what extent conducting MIRA leads to improvement of the innovation readiness of long-term care organizations. Additional instruments may be needed to help organizations develop more systematic and long-term approaches to improving innovation readiness (49, 50). Future research should include longitudinal studies to follow organizations that implement steps to improve innovation readiness and explore how these efforts contribute to successful innovation outcomes. Simultaneously, the applicability of MIRA across various long-term care settings, including disability care, maternity care, and rehabilitation services, warrants further research (51). A pilot study testing the comprehension and clarity of MIRA could be valuable. The materials developed for MIRA in long-term care (framework, questionnaire, and consensus meeting) are readily available for replication in these diverse contexts. Ultimately, driving progress in innovation readiness requires continued integration of research, practice, and policy (10, 24).

### Strengths and limitations

Several strengths and limitations of this study should be taken into consideration. First, this study was conducted across 10 diverse long-term care organizations (small, medium, and large) with a wide geographical spread in the Netherlands. Purposive sampling ensured variation in organizational size and context. The broad selection of healthcare professionals (representing different organizational levels, roles, and functions) was particularly appreciated by participants of the consensus meeting. Another strength of the study is the use of an online MIRA Questionnaire with a predefined structure for the MIRA Consensus meeting. MIRA was executed by one researcher (MH), who ensured consistency in implementation across all participating organizations. In addition, the combination of quantitative (Likert scale data) and qualitative (open-ended questions) methods enabled a nuanced evaluation of MIRA's feasibility in practice. As the study focused on Dutch long-term care organizations, the identified feasibility of MIRA may, therefore, differ for organizations in other healthcare sectors within or outside the Netherlands. Purposive sampling may have introduced selection bias in terms of organizational interest or readiness to innovate.

### Implications for practice and research

Purposive sampling may have introduced selection bias, as organizations interested in this study might score higher on innovation readiness factors. This means the results may not be representative of the experiences of organizations that are less focused on innovation. Consequently, perspectives from organizations with less interest in innovation might be missing. This restricts the applicability of the findings to other contexts, as the sample only represents a specific group of innovation-oriented organizations. This potential overrepresentation may have resulted in relatively higher overall maturity scores and a stronger emphasis on structured innovation processes than might be found in a more diverse sample. Therefore, more research is needed to understand how innovation readiness develops across a broader range of long-term care organizations. Future studies could use a broader selection of organizations, with both higher and lower levels of interest in innovation organizing, to make the results more widely applicable and to better reflect the diversity of long-term care.

The observed differences between domains, particularly between strategy and learning-related factors, highlight areas that may require additional attention. At the organizational level, management may integrate MIRA into their strategic plans to repeat the assessment annually, informing a systematic long-term strategy for innovation readiness. Embedding MIRA in regular planning processes may help organizations monitor progress over time. During the MIRA Consensus meetings, participants indicated an interest in repeating MIRA annually to monitor the development of their innovation readiness. For this purpose, MIRA should be further developed to detect changes in innovation readiness over time (52). Although organizations found the support of an external facilitator helpful in the consensus meetings, building internal capacity to conduct these could strengthen learning within the organization and make it easier to repeat the use of MIRA.

Based on participant feedback on the MIRA Questionnaire—specifically its terms, explanations, instructions, and design—the findings indicate promising conditions for broader-scale use of MIRA. To support annual, independent, and practical use of MIRA, we intend to develop an online version of the MIRA Questionnaire and facilitate broader implementation in long-term care, accompanied by a scoring interpretation guide and a facilitation guide for MIRA Consensus meetings (53, 54). We propose conducting interviews with future participants to assess the clarity, comprehension, acceptability (such as ease of administration), and suitability of the online version. For future use, MIRA may benefit from automating data analyses to improve efficiency, particularly when scaling up.

MIRA may have potential for broader application across different healthcare settings. Future research could further examine its clarity, usability, and acceptability among diverse participants to enable its applicability in various healthcare contexts. In addition, the conceptual foundation of MIRA could be strengthened by incorporating insights from validated multidimensional approaches that capture complex organizational factors and interventions (55, 56).

## Conclusion

In today's society, organizations need to be able to effectively organize “innovation” to sustain their contribution to high-quality care and working conditions in long-term care. To the best of our knowledge, the Maastricht Innovation Readiness Approach (MIRA)—which was iteratively developed in close collaboration with stakeholders in long-term care organizations—is one of the first instruments to enable healthcare organizations to self-assess their current state of innovation readiness. The feasibility study shows that MIRA enhances internal awareness and reflection, helps organizations identify areas for improvement, and supports more strategic steps toward strengthening innovation readiness. With further development and integration into everyday practice, MIRA presents a promising instrument for fostering innovation readiness in long-term care. MIRA can help organizations better navigate the complex challenges of innovation and contribute to more responsive, sustainable, and high-quality care. Future research should follow organizations over time to explore whether and to what extent conducting MIRA contributes to the improvement of innovation readiness across long-term care organizations.

## Data Availability

The datasets presented in this study can be found in online repositories. The names of the repository/repositories and accession number(s) can be found in the following: https://osf.io/x73p6/.
