# Supplementary material for: Feasibility of the Maastricht Innovation Readiness Approach: a self-assessment of innovation readiness in long-term care organizations for older adults
Source: Front Health Serv. 2026 Apr 30;6:1660216. doi: 10.3389/frhs.2026.1660216 (PMC13171825; doi:10.3389/frhs.2026.1660216)
Supplement: Supplementary file 1 [file Table1.docx]

Supplementary Table S1 Set-up MIRA Consensus meeting

This table provides a detailed agenda (time and content) of the set-up of the MIRA Consensus meeting.

| Agenda for Consensus Meeting | | |
| --- | --- | --- |
| 5 min | Introduction (brief) | What are your expectations? (Create name tags) |
| 5 min | Agenda Overview | Explanation of Objectives: 1) Understanding Innovation Readiness (IR), 2) Feedback on IR within your organization, 3) Reaching a shared understanding. The last objective may be too ambitious; the primary goal is to interpret the level of innovation maturity as a collective starting point. |
|  |  | Feedback on the Four Main and Sub-Domains.  Then, discussion of key findings within the main domains. |
| 5 min | Background / Research on the Questionnaire | Explanation of the questionnaire’s background / innovation readiness factors.  All factors of the Innovation Readiness Framework are essential.  Their interrelation is crucial; you cannot innovate effectively by implementing only half of the factors. |
| 15 min | Feedback Session | Per (sub)domain at the organizational level.  Identify notable observations, such as wide variation, lack of visibility, or differing levels of maturity within subfactors of a domain. |
| 40 min | Discussion of Domains with Large Variance | Discussion of key points regarding the variance in results.  Purpose of the discussion:  How did this happen?  What impact does this have on innovation?  Why do opinions differ strongly on certain factors?  A. Why do we have a lot of agreement on certain factors? For example, if something is well-organized, ask for concrete examples.  B. On the factors where we score lower:  What is the urgency? Is it causing problems?  Ask about the background; for example, are there no agreements made?  What evidence is there in terms of policy and actions? |
| 10 min | Determining the General and Domain-Specific Level of Innovation Readiness | Consensus Discussion  Is reaching consensus desirable?  Discussion on potential gaps in knowledge, communication, or development stage Insights from the collective assessment of innovation readiness. |
| 10 min | Evaluation and Next Steps | Reflection on the MIRA approach.  Completion of the paper evaluation  Discussion on action perspectives.  What do you need for the next steps?  Work session / Brainstorm/ next steps on the four domains / priorities |

| Participants | Representation of participants who completed the questionnaire  Diversity: Strategic/tactical/operational – caregivers/support staff/medical |
| --- | --- |
| Setting | Conducted at the long-term care organization |
| Execution | Discussion with the coordinator.  Results sent to the coordinator in advance for further distribution.  Results presented with follow-up questions for reflection.  Process facilitation in consultation with the care organization’s coordinator. |
| Objectives (from a research perspective) | Collect Participant Reflections:   - feedback on the quality and process of the questionnaire and the consensus meeting. - Evaluate Against MIRA Goals/Assess how well the process aligns with the objectives of MIRA |
| Steps consensus meeting | - Present findings from self-assessment - Engage in dialogue to interpret the results - Aim for a shared understanding of the organization’s position on innovation readiness. |
